# Delivery of *BikDD* proapoptotic gene in Peptide-18-targeted Poly(2-oxazoline)-DOPE nanoliposomes for breast cancer models

**DOI:** 10.55730/1300-0152.2706

**Published:** 2024-09-05

**Authors:** Zeynep Büşra BOLAT, Ayça Ece NEZİR, Ongun Mehmet SAKA, Itır Ebru ZEMHERİ, Sevgi GÜLYÜZ, Umut Uğur ÖZKÖSE, Özgür YILMAZ, Asuman BOZKIR, Dilek TELCİ, Fikrettin ŞAHİN

**Affiliations:** 1 Department of Genetics and Bioengineering, Faculty of Engineering, Yeditepe University, Ataşehir, İstanbul, Turkiye; 2 Department of Molecular Biology and Genetics, Hamidiye Institute of Health Sciences, University of Health Sciences-Türkiye, İstanbul, Turkiye; 3 Experimental Medicine Research and Application Center, University of Health Sciences İstanbul, Turkiye; 4 Department of Pharmaceutical Technology, Faculty of Pharmacy, Ankara University, Ankara, Turkiye; 5 Department of Pathology, Umraniye Training and Research Hospital, İstanbul, Turkiye; 6 Materials Institute, Marmara Research Center, TÜBİTAK, Gebze, Turkiye; 7 Department of Chemistry, Faculty of Science and Letters, İstanbul Technical University, İstanbul, Turkiye; 8 Department of Chemistry, Faculty of Science and Letters, Piri Reis University, İstanbul, Turkiye

**Keywords:** Breast cancer, BikDD gene, targeted therapy, gene delivery, nanoliposomes

## Abstract

Breast cancer is one of the most common cancers and a significant cause of death in females worldwide. For effective breast cancer treatment, using systems with a promising delivery of anticancer agents is an important strategy. Peptide 18 (P18), a tumor-homing peptide, shows a high binding affinity toward breast cancer cells. Nanoliposomes are known to have enhanced accumulation ability in tumors with longer systemic circulation. In this study, Poly (2-ethyl-2-oxazoline) (PEtOx) polymers conjugated with DOPE are used to prepare PEtOx-DOPE nanoliposomes. *BikDD*, a mutant form of the Bik gene and a member of the BH3-only proapoptotic genes, mimics the constitutively phosphorylated form of the gene. To the best of our knowledge, this study presents a novel approach by investigating P18-conjugated PEtOx-DOPE nanoliposomes (P18-PEtOx-DOPE) for the targeted delivery of *BikDD* to the AU565 breast cancer model. A site-directed mutated *BikDD* was loaded into P18-PEtOx-DOPE nanoliposomes, and the targeted drug delivery system was assessed in in vitro and in vivo breast cancer models for efficiency, safety, and efficacy. The increased Bik mRNA expression levels in AU565 cells suggest a high effectiveness of the targeting PEtOx-DOPE nanoliposomes. Following the in vitro studies, the delivery of *BikDD* by P18-PEtOx-DOPE nanoliposomes was analyzed in CD-1 nude mice models. The animal study showed no significant difference in the tumor volume of the CD-1 nude mice treated with P18-PEtOx-DOPE-BikDD nanoliposomes compared to the free delivery of *BikDD*. Our preclinical studies suggest that P18-PEtOx-DOPE-BikDD nanoliposomes may be promising gene carriers for targeted breast cancer therapy. Thus, further studies should be carried out to determine the prolonged use of this drug delivery system in breast cancer therapy.

## 1. Introduction

Breast cancer is one the most common cancers, with a nearly 25.1% incidence rate worldwide. Almost 20%–25% of breast cancers show overexpression of the human epidermal growth factor receptor 2 (HER2) (Arteaga et al., 2011). The HER2-positive breast cancer subtype is aggressive compared with HER2-negative tumors and shows poor response to standard chemotherapy (Li et al., 2017). Thus, target-specific therapeutic methods are needed as effective anticancer treatments. Gene therapy is one approach that has shown potential for treating breast cancer in recent years.

The Bcl-2 family is the primary regulator of the mitochondrial apoptosis pathway and is classified into three subgroups according to their Bcl-2 homology (BH) domains: the multidomain antiapoptotic, the multidomain proapoptotic, and the BH3-only proapoptotic. The *Bik* gene is a member of the BH3-only proapoptotic subgroup (Certo et al., 2006; Suvarna et al., 2019) and has been proposed as an apoptosis-potentiating therapeutic gene in cancers (Zou et al., 2002). *BikDD* is a constitutively active mutant form of the proapoptotic *Bik* gene in which mutations in T33D and S35D were made to mimic the constitutively phosphorylated form of the gene, thus enhancing binding affinity to its multiple binding partners (Lang et al., 2011). Studies have shown that *BikDD* effectively induces apoptosis and inhibits breast (Lang et al., 2011), lung (Sher et al., 2009), pancreatic (Xie et al., 2007), and prostate (Xie et al., 2014) cancers. Furthermore, systemic delivery of *BikDD* under the control of a breast-specific promoter for gene expression in nanoparticles significantly suppressed tumor growth in triple-negative breast cancer mouse models (Xie et al., 2012). Therefore, the targeted delivery of proapoptotic genes by nanoparticles shows promise in cancer treatment.

Poly (2-ethyl-2-oxazaoline) (PEtOx) is a polymer that is biocompatible and soluble, varies in size, and possesses chemically functional properties (Luxenhofer et al., 2012). In the last decade, more attention has been paid to polycationic gene delivery vectors because they are easily prepared, have a versatile chemical structure, and lack immune response in vivo (Zhao et al., 2017). Nanoliposomes, made of a lipid bilayer, are used as nanocarriers to encapsulate active agents; they are advantageous due to their increased solubility and prevention of premature release into the bloodstream. These nanocarriers are safe and efficient drug delivery systems because of their biodegradability and biocompatibility (Pourmadadi et al., 2023). DOPE is a neutral phospholipid that can alternate structurally by pH change and shows low toxicity and high stability in the delivered particle. As a helper cationic colipid, DOPE increases transfection activity and has been shown to have the same transfection efficacy as the commercially available transfection reagent Lipofectamine 2000 when mixed with cationic lipid (Masotti et al., 2009; Mochizuki et al., 2013). Modifying the surface of nanoparticles can overcome the low specificity and toxicity problems of anticancer agents, leading to better therapeutic strategies for patients (Gholami et al., 2024). Peptides (Shahin et al., 2013), antigens (Pourmadadi et al., 2024), and aptamers (Kim et al., 2022) are used for detecting breast cancer and can be conjugated on nanoliposomes for use as targeting agents.

Peptide 18 is a tumor-homing peptide showing high binding affinity to breast cancer cells compared to healthy mammary epithelium cells (Soudy et al., 2011). We previously showed that Peptide18 conjugated to PEtOx-based polymersome (Oz et al., 2021) and that nanoliposome (Bolat et al., 2021; Devrim et al., 2021) nanocarriers effectively target breast cancer AU565 cell lines.

In this study, the targeted delivery potential of proapoptotic *BikDD* encapsulated to the P18-PEtOx-DOPE (PPD-BikDD) nanoliposome was examined on breast cancer AU565 cell lines. Transfection of *BikDD* via PPD-BikDD nanoliposome led to an increase in *Bik* gene expression levels in the AU565 cell lines. Consequently, PPD-BikDD nanoliposomes displayed a significant decrease in the cell viability of AU565 breast cancer cells. The in vivo experiments demonstrated that the AU565 CD-1 nu/nu mice model treated with PPD-BikDD exhibited a significantly greater inhibitory effects on tumor growth compared to the group treated with the free *BikDD* gene, suggesting that targeted delivery of *BikDD* enhances its anticancer activity.

## 2. Materials and methods

### 2.1. Materials

QuikChange Lightning Site-Directed Mutagenesis Kit (Agilent Technologies, Santa Clara, CA, USA), Cell proliferation reagent WST-1 (Roche, Basel, Switzerland), Trizol Reagent (Thermo Fisher Scientific, Waltham, MA, USA), Sensiscript RT Kit (Qiagen, Hilden, Germany), QuantiTect SYBR Green PCR Kit (Qiagen, Hilden, Germany), and Matrigel, supplied by Corning (Corning, NY, USA), were used in the experiments.

### 2.2. Site-directed mutagenesis

The QuikChange Lightning Site-Directed Mutagenesis Kit manufacturer’s protocol was used for site-directed mutagenesis. Threonine 33 and Serine 35 of pEGFP-Bik plasmid (Addgene; cat #10952) were changed to Aspartic Acid (D) using *5’-GTTCTTGGCATGGACGACGATGAAGAGGACC-3’* and *5’-GGTCCTCTTCATCGT-CGTCCATGCCAAGAAC-3’* primers to obtain mutated pEGFP-BikDD (BikDD) plasmid. The sequences of the BikDD mutant construct were confirmed using automated sequencing (Figure S1).

### 2.3. Preparation of P18-PEtOx-DOPE-BikDD nanoliposomes

Cationic polymer PetOx-DOPE conjugated with tumor-homing peptide P18 [Cystein]-WXEAAYQRFL was synthesized by click reactions, as described previously (Gulyuz et al., 2020). P18-PEtOx-DOPE (PPD) polymers were formulated into cationic PPD nanoliposomes and prepared with the lipid hydration method, as previously described (Saka and Bozkir, 2018). Briefly, L-α-phosphatidylcholine (Sigma-Aldrich, St. Louis, MI, USA), cholesterol (Sigma), and the synthesized polymer (140:40:20 mg w/w/w) were dissolved in 15 mL of ethanol. The well-dissolved solution was placed in a round-bottom flask, and the ethanol was evaporated under a pressure of 200 mbar for 15 min using a rotary evaporator. Precisely 20-mL ultra-pure bidistilled water containing BikDD plasmid (1:2 molar ratio) was added to the dry film and rotated for hydration at 50 °C overnight. To reduce and optimize the liposome size, the resulting formulated solution was transferred to the extruder (IKA, Staufen im Breisgau, Germany). A 20-mL formulation was diluted with 50-mL bidistilled water and exposed to the extruder for 25 min at a pressure of 1000 mbar at 250 rpm. After obtaining 100-nm-sized unilamellar liposomes, we lyophilized the formulation; in the subsequent studies, formulations were reconstituted at desired concentrations.

### 2.4. Cell culture

Breast cancer AU565 (CRL-2351) cell lines were obtained from the American Type Culture Collection (USA); they were cultured in RPMI 1640 medium supplemented with 10% fetal bovine serum (FBS, Thermo Fisher Scientific, Waltham, MA USA), 100 U/mL of penicillin, and 100 μg/mL of streptomycin (1% PS, Thermo Fisher Scientific). The cells were maintained at 37 °C in a humidified atmosphere at 5% CO_2_.

### 2.5. Quantitative real-time PCR

For the quantitative real-time PCR (qRT-PCR) analysis, total RNA was isolated from AU565 cells treated with empty PetOx-DOPE (PD) and PPD-BikDD nanoliposomes using Trizol following the manufacturer’s protocol. The RNA was transcribed into cDNAs using Senscript kit, and real-time PCR was performed using SYBR as described in an earlier study (Oz et al., 2020). The *Bik* primers used in this study were forward:5’-*GAGACATCTTGATGGAGACC*-3’ and reverse:5’-*TCTAAGAACATCCCTGATGT*-3’. The 18SrRNA (Qiagen, Hilden, Germany) reference gene was used as a housekeeping gene to ensure equal loading, and the data were analyzed using 18SrRNA as a normalization control.

### 2.6. Cell viability assay

The cytotoxic effect of free BikDD, PPD-BikDD, and PD nanoliposomes in the AU565 cells were evaluated using cell proliferation reagent WST-1 assay. The AU565 cells, seeded at a density of 1 × 10^5^ cells/well, were treated the following day with free BikDD, PD, and PPD-BikDD nanoliposomes. The free BikDD was delivered by commercial transfection reagent Lipofectamine 2000, following the manufacturer’s protocol. WST-1 was used according to the manufacturer’s protocols, and after 60 min incubation, the absorbance values were measured at 450 nm in a microplate reader (ELx800, BioTek, Winooski, VT, USA). The cell viability percentage was calculated by assigning nontreated cells an absorbance value of 100%.

### 2.7. Serum stability

After preparing the PPD nanoliposomes, they were stored for 2 weeks in phosphate-buffered saline (PBS) buffer in amber vials at 4 °C. The serum stability assays were performed by incubating the nanoliposome formulation in 3 mL Dulbecco’s Modified Eagle’s Medium (DMEM) supplemented with 10% FBS for 24 h at 37 °C. More specifically, 60 μL of the formulation was added to the DMEM at a 50:1 ratio with no further agitation. The analysis of size distribution and zeta potential values evaluated the effect of the serum on the liposomes (Jones and Nicholas, 1991; Chan et al., 2009).

### 2.8. In vivo antitumor activity

For the in vivo anticancer activity assay, athymic CD-1 nu/nu female mice (5–6 weeks old, 20 ± 2g) obtained from Charles River Laboratories (Germany) were housed in sterilized and filtered cages and subjected to a constant temperature and relative humidity of 23 ± 1 °C and 60 ± 10%, respectively. The mice were kept for 12 h in dark/light environmental conditions, fed special diets for nude mice, and given water ad libitum. Animal procedures were approved by the Institutional Animal Care and Welfare Committee of Yeditepe University in Türkiye (approval no. #644). To obtain orthotopic breast cancer models, 8 × 10^6^ AU565 cells with a 1:1 ratio of Matrigel were injected into the second mammary fat pad of CD-1 nu/nu female mice, as suggested in a previous study (Bolat et al., 2021). Following the third day of inoculation, the mice were injected intraperitoneally once every 3 days with vehicle (PBS) control, PD nanoliposome, PPD-BikDD nanoliposome (5 μg DNA), or free BikDD (5 μg DNA). The tumor size and weight of each animal were measured before every injection, and the mice were examined for morbidity and mortality until the end of the study. For the histopathology and immunohistochemical examinations, the animals were sacrificed using the cervical dislocation method after 27 days. The tumor volume (mm^3^) was calculated after the resection of tumors using the following formula: tumor volume = ½(length × (Width)²) (Huang et al., 2015). Organs and tumor tissues fixed in 10% formalin were paraffinized and processed for hematoxylin and eosin (H&E) staining at the Department of Pathology at Umraniye Training and Research Hospital.

### 2.9. Statistical analysis

All data in this study was represented as mean ± standard deviation (SD) values. Parametric statistics between two groups were analyzed using Student’s t-test; for multiple comparisons, ANOVA was used. GraphPad Prism Version 6 for Windows (trial version, GraphPad Software, San Diego, CA, USA) was used to perform statistical analysis, and differences were considered significant at p ≤ 0.05 (*), p ≤ 0.01 (**), p ≤ 0.001 (***), and p ≤ 0.0001 (****).

## 3. Results

### 3.1. Serum Stability in the PPD-BikDD nanoliposomes

The unilamellar vesicles were successfully prepared using a newly synthesized polymer prepared by our research team (Gulyuz et al., 2020). Formulation dimensions were approximately 100 nm, and the BikDD plasmid encapsulated formulation had a negative charge. To investigate the interaction of the liposomes in the presence of amino acids and fetal serum, particle size, and zeta potential measurements were performed using an aqueous dip cell in the automatic mode with a Zetasizer Nano ZS (Malvern Instruments, Malvern, UK) (Saka et al. 2023). The particle size and zeta potential values varied due to the interaction of the nanoliposomes with serum components; these variations are summarized in the Table. There was no significant change (p > 0.05) in particle size and zeta potential value between the freshly prepared formulation and the reconstituted formulation kept in the refrigerator for 2 weeks. This shows that both formulations were stable for at least 2 weeks at 5 ± 3 °C. In addition, no significant change in the size of PPD nanoliposome formulations after incubation with serum was found.

### 3.2. PPD-BikDD increased Bik gene expression in the AU565 cell lines

**Figure 1:**
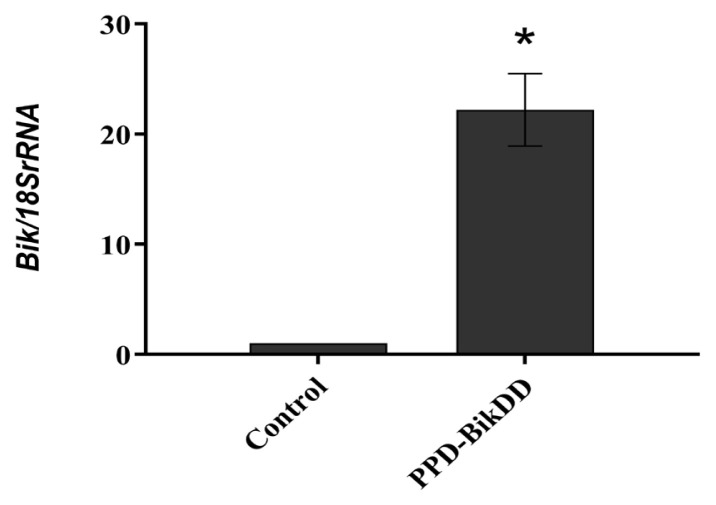
Fold increase of relative Bik/18SrRNA expression using qRT-PCR in AU565 cell lines treated with PPD-BikDD nanoliposomes and nontreated AU565 cells (control). Data represents the average of the three independent experiments ± SD (*p ≤ 0.05).

The relative Bik/18SrRNA mRNA expression of the AU565 cells treated and nontreated with PPD-BikDD were analyzed using qRT-PCR. The results show that the AU565 cells treated with PPD-BikDD expressed the *Bik* gene nearly 22 times more than the untreated cells (Figure 1). These results confirm that PPD-BikDD effectively increases proapoptotic *Bik* gene expression in AU565 cell lines.

### 3.3. Effect of P18-PEtOxDOPE-BikDD nanoliposome on cell viability

**Figure 2:**
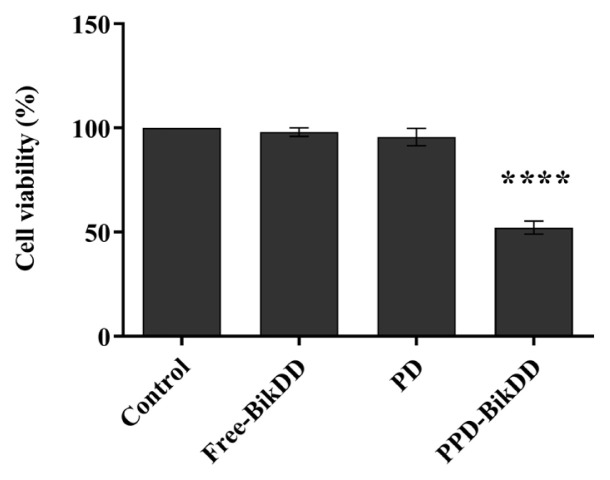
Determination of the cytotoxicity effect of PPD-BikDD on AU565 cells. AU565 cells were treated with PD, PPD-BikDD formulation, and free *BikDD* for 72 h. Cell viability was assessed by measuring the absorbance change using a microplate reader at 450 nm. Data represents the average of the three independent experiments ± SD (****p ≤ 0.001).

The therapeutic effect of *BikDD* delivered by the PPD-BikDD nanoliposomes and Lipofectamine 2000 reagent (free *BikDD*) to the AU565 cells were analyzed using WST-1 cell proliferation assay. The AU565 cells were subjected to free *BikDD*, PD, and PPD-BikDD nanoliposomes for 72 h. The PPD-BikDD nanoliposomes caused a significant reduction in cell viability to the AU565 cells (52.16%) compared to the nontreated cells, whereas PD and free *BikDD* did not show a significant decrease in cell viability compared to the nontreated AU565 cells (Figure 2).

### 3.4. Therapeutic efficacy of P18-PEtOx-DOPE-BikDD in an orthotopic breast cancer model

**Figure 3:**
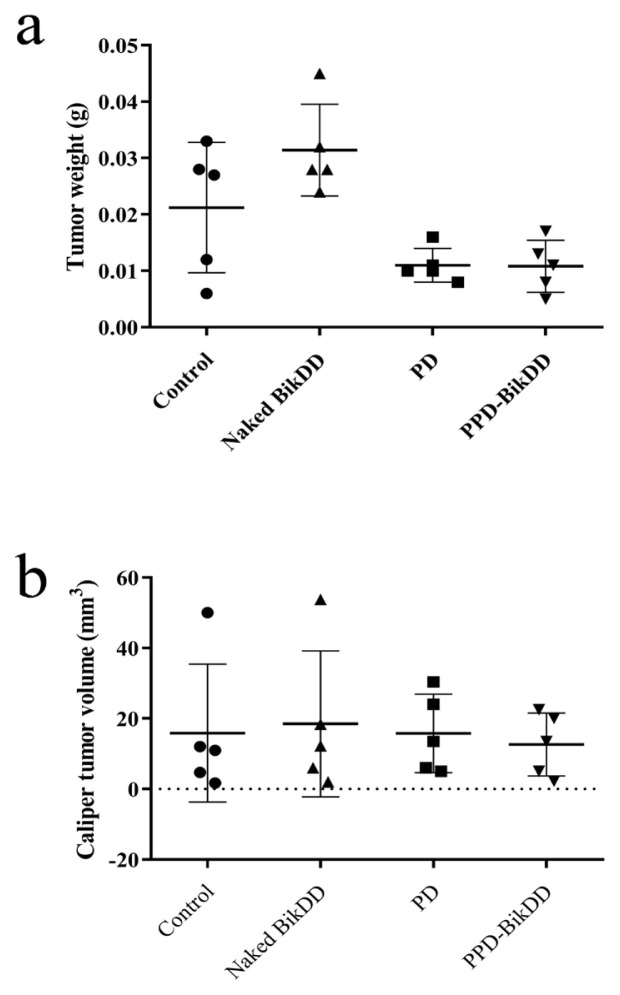
Antitumor effect of PPD-BikDD nanoliposomes on CD-1 nude mice models with the graph showing the distribution of the isolated (a) tumor weights and (b) tumor volumes of CD-1 nude mice in four groups subjected to treatment with either vehicle control (PBS), naked *BikDD*, PD, or PPD-BikDD nanoliposomes.

A breast cancer orthotopic CD1 nu/nu mouse model was established using AU565 cells to elucidate the drug delivery efficiency of PPD-BikDD nanoliposomes. The antitumor effect of P18-PEtOx-DOPE-BikDD in vivo was investigated using CD1 nu/nu female mice bearing AU565 orthotopic xenografts. Each group was treated intraperitoneally with either a control vehicle (PBS), PEtOx-DOPE nanoliposomes, P18-PEtOx-DOPE-BikDD nanoliposomes, or 5 μg of naked *BikDD*. The average body weight of mice recorded on the 5th and 7th injections showed no weight loss in all treatment groups, suggesting tolerable treatment dosage (Figure S2). Tumors grew progressively and rapidly in the mice treated with control and PD nanoliposome cohorts (Figure S3). The tumor weight and volume of PPD-BikDD nanoliposome cohorts decreased compared to the control cohort despite no statistical significance (Figure 3a–b).

**Figure 4:**
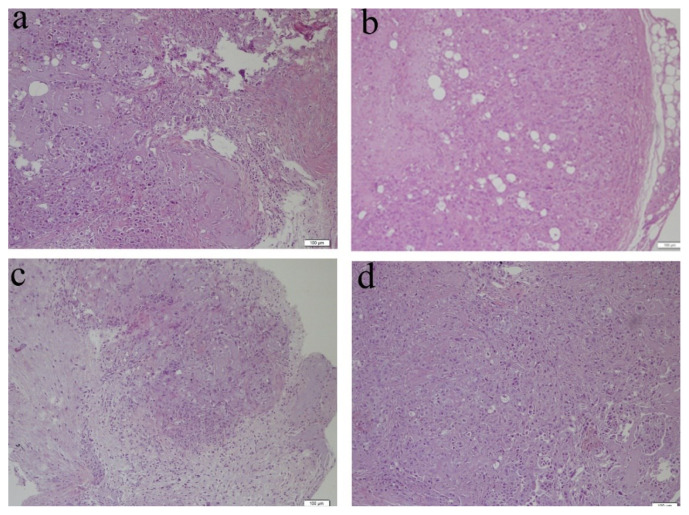
Histopathological analysis of the tumor samples of PPD-BikDD liposomes. H&E staining was used to stain the tumors isolated from CD-1 nude mice in four groups subjected to the treatment of either (a) vehicle control, (b) naked *BikDD*, (c) PPD-BikDD nanoliposomes, or (d) PD nanoliposomes; scale bar: 100 μm.

Following the sacrifice and tumor isolation of the animals, samples were stained by H&E for histopathological analysis. All tumor types were characterized as adenocarcinoma, verifying the successful generation of the AU565 orthotopic CD-1 nude mouse models. Histological and nuclear grading of all tumors in the four different treatment groups were recorded as Grade 3 (Table S). The results show that histopathological examination of tissue from PPD-BikDD nanoliposomes showed necrosis in tumor sections compared to tissues of the control and PD cohorts (Figure 4).

## 4. Discussion

Breast cancer is a significant health problem and one of the leading causes of death in females worldwide. According to the clinical and pathological tumor profile of breast cancer patients, treatment approaches include hormonal therapy, radiotherapy, chemotherapy, or surgery (Moo et al., 2018).

However, breast cancer recurrence is still a major problem, and novel site-specific delivery strategies for the preferential delivery of anticancer agents are required. Active tumor targeting modality is an emerging and indispensable platform for safe and efficient cancer treatment. This method maximizes therapeutic efficacy and minimizes the adverse effects (Zhong et al., 2014; Morales-Cruz et al., 2019). Tumor-homing peptides are small, have enhanced tissue penetration capacity, and can be easily conjugated to drugs conferring low immunogenicity (Ruoslahti, 2017; Hossein-Nejad-Ariani et al., 2019). P18, a tumor-homing peptide, plays a vital role in many cancers (Collard et al., 2001; Chuang and Huang, 2007; Tang et al., 2012). Therefore, incorporating active targeting moieties such as P18 onto nanocarrier formulations increases the specificity and penetration of a drug within the tumor and enables the nanocarriers to selectively bind to the target receptor that is overexpressed in breast cancer cells. In an earlier study, we investigated the potential of the binding affinity of PPD nanoliposomes to AU565 cells (Saka et al., 2023). PPD nanoliposomes showed a strong binding affinity to AU565 cells compared to normal epithelial breast cells (Devrim et al., 2021; Saka et al., 2023), making this nanocarrier a candidate drug carrier that can be used in breast cancer therapy. The toxicity to normal cell lines is a major concern in targeted nanocarrier-based drug delivery systems (Hossen et al., 2019). Our earlier cytotoxicity experiments showed that PD lipopolymer and nanoliposome formulations exhibit no significant cytotoxicity on normal epithelial breast MCF10A cells (Bolat et al., 2021), HUVEC (Gulyuz et al., 2020), mesenchymal stem cells, and kidney HEK293 cells (Devrim et al., 2021). This study demonstrated that empty nanoliposomes are not significantly toxic to AU566 cell lines.

Most nanoliposomes designed for therapeutic purposes are between 50–100 nm in diameter to avoid phagocyte uptake and achieve prolonged blood circulation time (Danaei et al., 2018), and our PPD nanoliposomes were within this range. Several authors reported that serum proteins do not affect small unilamellar vesicle liposomes in the range of 40–400 nm at low serum concentrations. However, at high concentrations, large-sized liposomes were found not intact (Jones and Nicholas, 1991). As a result, the hydrodynamic diameter formed by PEtOx and lecithin (phosphatidylcholine) suggests that its structure is largely preserved and that plasma protein binding was not an important factor in the present formulations. Furthermore, when the formulations of zeta potentials were analyzed, a significant change toward a positive value occurred. Although the zeta potential value increased when compared to the charge of the serum medium, no significant changes were observed. As the serum medium was applied 50 times more in volume than the formulations, it was concluded that the zeta potential value did not change according to the concentration of the serum environment. The released BikDD plasmid into the medium was also determined to bind to serum proteins, maintaining the current negative charge. Several authors (2011) reported that their formulations, prepared with different lipoplexes (Li et al., 2011), also showed similar Zeta potential values independent of lipid structure with the serum environment. The same authors also emphasized that serum strongly affects Zeta potential but protects the structure, depending on the lipid ratio. According to the data and the results obtained in similar studies, it has been concluded that our formulations preserve their structure in DMEM with FBS.

The *Bik* gene is a member of the BH 3-only proapoptotic subgroup and is used as an apoptosis-potentiating therapeutic gene in cancers. Apoptosis mediated by the *Bik* gene is dependent on the *Bax* gene (Certo et al., 2006), and studies have shown that *Bik* enhanced apoptosis in T-lymphoma cells resistant to corticosteroid (Daniel et al., 1999). Nonviral gene delivery of the *Bik* gene significantly inhibited tumors in human breast cancer mouse models (Zou et al., 2002). *BikDD* is a constitutively active mutant form of the proapoptotic *Bik* gene, in which mutations in T33D and S35D were made to mimic the constitutively phosphorylated form of the gene, thus enhancing its binding affinity to its multiple binding partners. *BikDD* effectively induces apoptosis and inhibits breast cancer (Lang et al., 2011), and systemic delivery of *BikDD* by targeted nanoparticles showed significant suppression of tumor growth in breast cancer mouse models (Xie et al., 2012). Thus, the delivery of *BikDD* to breast cancer cells by targeted liposomes is a promising method in breast cancer therapy. Using a similar approach, in this study, we investigated the delivery of *BikDD* using PPD nanoliposomes for breast cancer therapy. Our results show that *BikDD* delivered by PPD showed a significant increase in *Bik* expression levels compared to the untreated group (Figure 1). A high transfection efficiency of nanocarrier-based DOPE formulations is preferred because of the fusogenic effect of the nonlamellar phase (Hafez et al., 2001). Recent studies have reported that DOPE transfection efficiency and internalization are cell-line dependent (Douglas et al., 2008; Izumisawa et al., 2011). Thus, delivery of *BikDD* by PDD nanoliposomes might be an effective drug delivery method.

Studies have shown that *BikDD* transfected to breast cancer MDA-MB-468 cells induced apoptosis by 40% to 80 % more than what was possible with the wild-type *Bik* (Lang et al., 2011). In the present study, we consistently showed that PPD nano liposome formulations successfully delivered *BikDD* to the AU565 cells, which resulted in a significant decrease in the cell viability of the AU565 cells in a dose-dependent manner (Figure 2); this suggests that the P18-PEtOx-DOPE liposome could be an excellent nanocarrier system to be tested in in vivo studies. We also investigated the antitumor effect of proapoptotic *BikDD* gene-loaded P18-PEtOx-DOPE nanoliposomes on AU565 tumor models. Our results show a decrease in tumor weight and volume in the PPD-BikDD nanoliposomes cohorts compared to the controls. However, this decrease was not significant, possibly due to the low dose of BikDD given per mouse because of the low loading capacity of PPD liposomes (5 μg of DNA/mice). In a previous study, several researchers showed a strong antitumor response on breast cancer MCF7 nude mice models by delivering BikDD using SN liposomes (15 μg of DNA/mice) (Li et al., 2003), suggesting that delivering a higher gene dosage was essential for an antitumor response.

In conclusion, this study describes a targeted therapeutic platform for the delivery of proapoptotic *BikDD* intracellularly to breast cancer AU565 cells. PPD-BikDD nanoliposomes exhibited high *Bik* gene expression levels in the AU565 cell lines, which resulted in the decreased cell viability of the AU565 cells at 72 h. The orthotopic xenograft model showed a decrease in tumor weight and volume of PPD-BikDD-treated CD-1 nu/nu female mice compared to the control cohorts. The particle size and zeta potential measurements revealed no significant change after weeks of incubation. This suggests PPD-BikDD nanoliposomes were stable in serum, which means they can potentially be used as therapeutic agents. Overall, this study on PPD-BikDD nanoliposomes can guide future research on targeted gene delivery systems for breast cancer therapy.

## Supplementary Data

Figure S1 Chromatogram results of pEGFP-BikDD vector. The black arrows indicate the site of mutation T→D and S→D.

Figure S2 Antitumor effect of PPD-BikDD liposomes on CD-1 nude mice body weight.

Figure S3 Antitumor effect of PPD-BikDD nanoliposomes on CD-1 nude mice tumors. Pictures represent the tumor isolated from CD-1 nude mice in four groups subjected to treatment with either vehicle control (PBS), PD nanoliposomes, naked *BikDD*, or PPD-BikDD liposomes.

**Table S:** Effect of PPD-BikDD liposomes on tumor tissues of CD-1 nude mice used in the study.

	Animal	Tumor Type	Histologic/Nuclear Grading
Control	1	Adenocarcinoma	H3/N3
	2	Adenocarcinoma	H3/N3
	3	Adenocarcinoma	H3/N3
	4	Adenocarcinoma	H3/N3
	5	Adenocarcinoma	H3/N3
PD	1	Adenocarcinoma	H3/N3
	2	Adenocarcinoma	H3/N3
	3	Adenocarcinoma	H3/N3
	4	Adenocarcinoma	H3/N3
	5	Adenocarcinoma	H3/N3
Naked BikDD gene	1	Adenocarcinoma	H3/N3
	2	Adenocarcinoma	H3/N3
	3	Adenocarcinoma	H3/N3
	4	Adenocarcinoma	H3/N3
	5	Adenocarcinoma	H3/N3
PPD- BikDD	1	Adenocarcinoma	H3/N3
	2	Adenocarcinoma	H3/N3
	3	Adenocarcinoma	H3/N3
	4	Adenocarcinoma	H3/N3
	5	Adenocarcinoma	H3/N3

## Figures and Tables

**Table:** Particle size and zeta potential values of BikDD plasmid-loaded PPD nanoliposomes (n = 3).

	Before incubation		After incubation	
	Particle size (nm)	Zeta potential (mV)	Particle size (nm)	Zeta potential (mV)
**PPD liposome**	93.34 ± 2.6010	–25.0 ± 0.4580	93.16 ± 0.5032	–10.7 ± 1.0600
**DMEM with 10% FBS**	-	-	148.0 ± 0.2082	–13.6 ± 1.0000
