# Supplementary figures and images for: Delivery of BikDD proapoptotic gene in Peptide-18-targeted Poly(2-oxazoline)-DOPE nanoliposomes for breast cancer models
Source: Turk J Biol. 2024 Sep 5;48(5):299–307. doi: 10.55730/1300-0152.2706 (PMC11518345; doi:10.55730/1300-0152.2706)

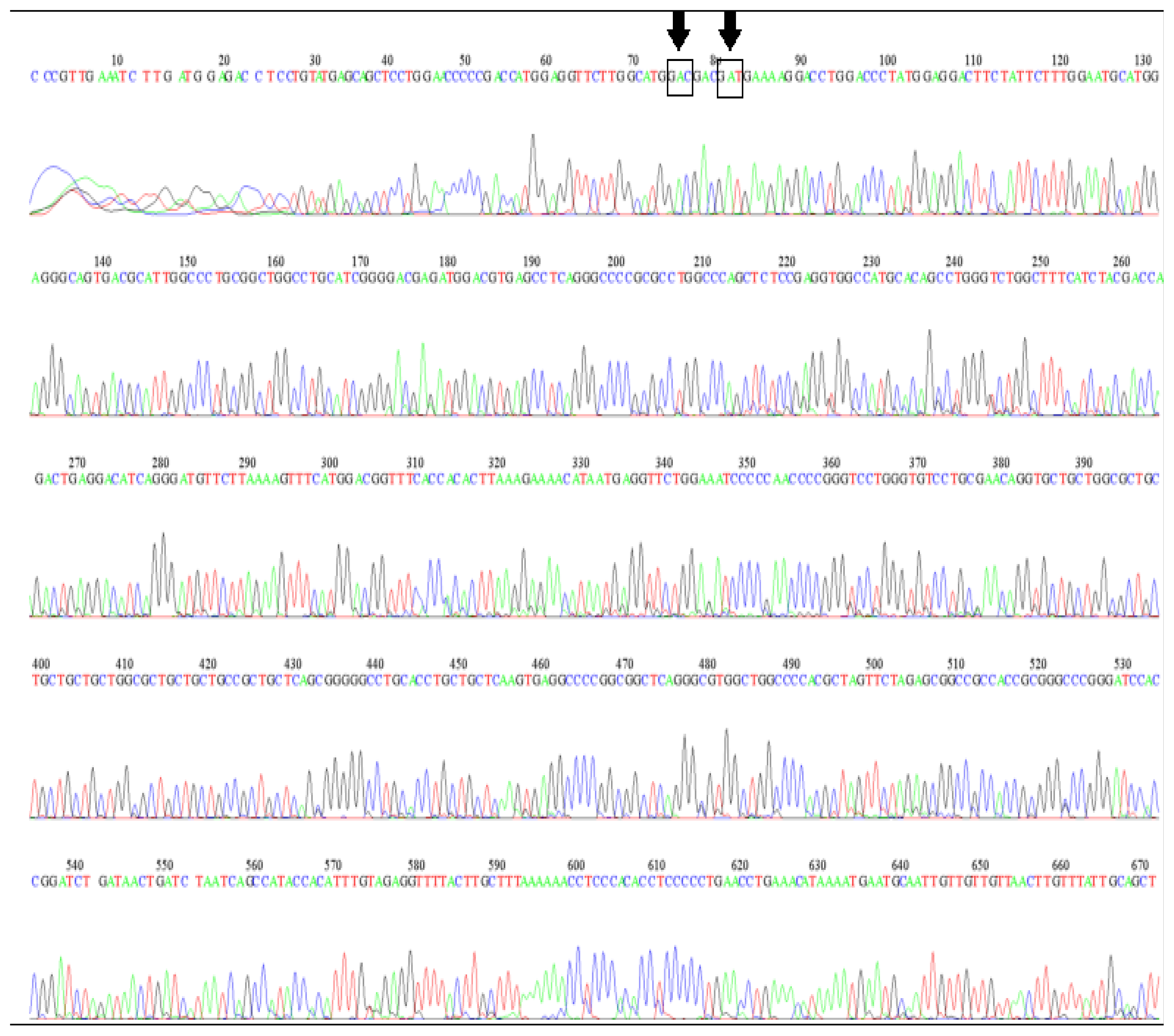

Supplement: Figure S1 — Chromatogram results of pEGFP-BikDD vector. The black arrows indicate the site of mutation T→D and S→D. [file tjb-48-05-299s1.tif]

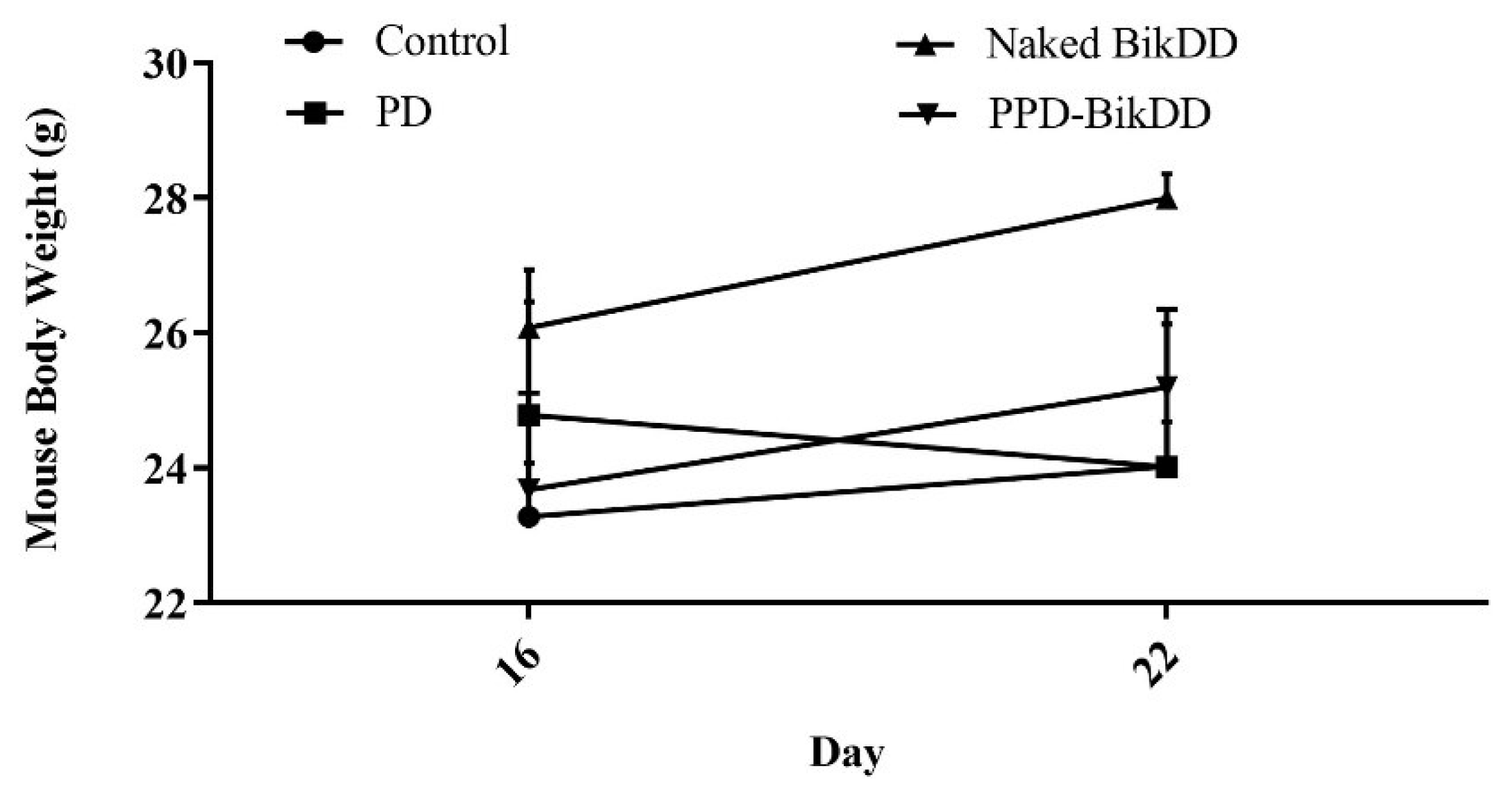

Supplement: Figure S2 — Antitumor effect of PPD-BikDD liposomes on CD-1 nude mice body weight. [file tjb-48-05-299s2.tif]

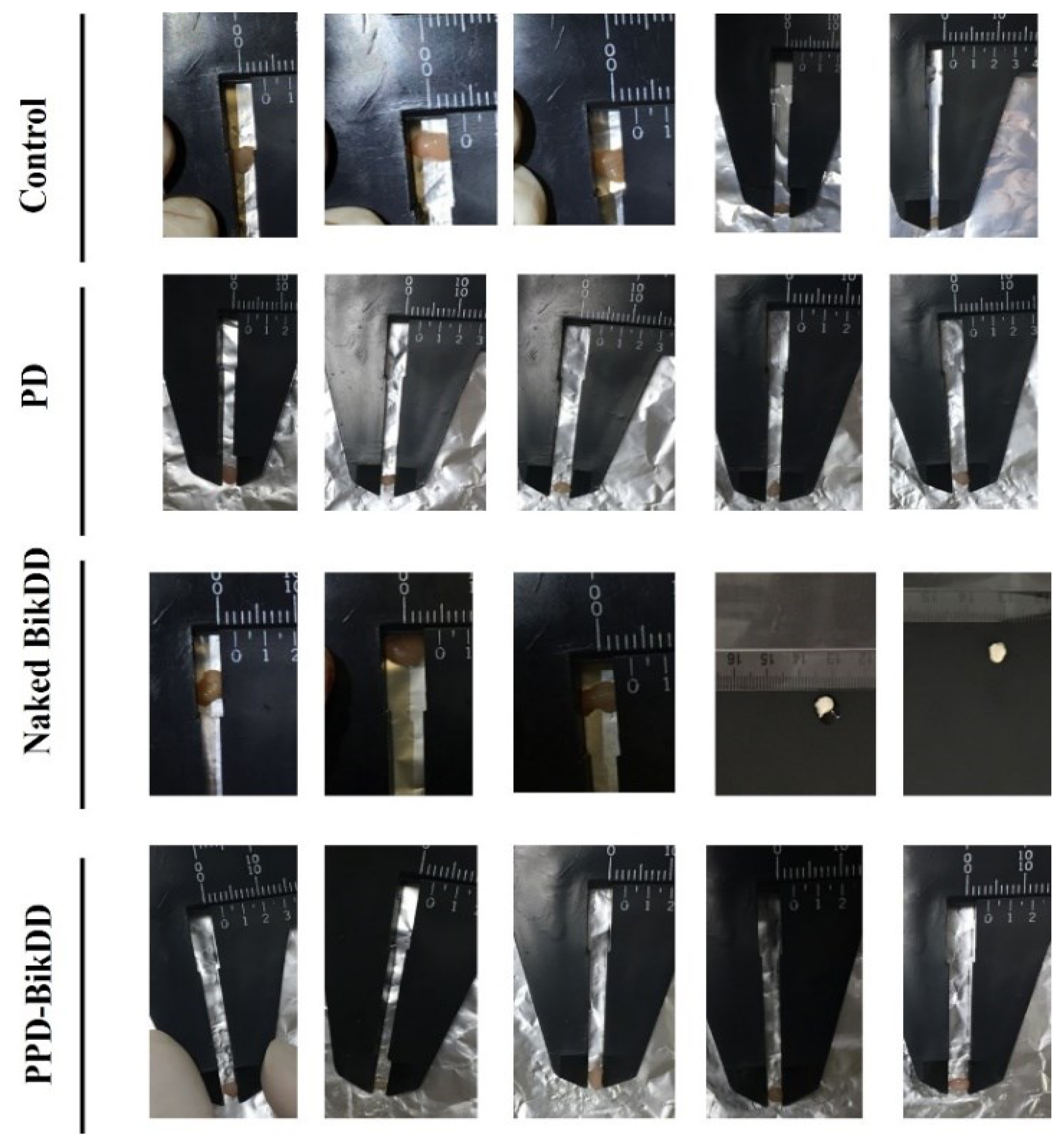

Supplement: Figure S3 — Antitumor effect of PPD-BikDD nanoliposomes on CD-1 nude mice tumors. Pictures represent the tumor isolated from CD-1 nude mice in four groups subjected to treatment with either vehicle control (PBS), PD nanoliposomes, naked BikDD, or PPD-BikDD liposomes. [file tjb-48-05-299s3.tif]
